# Effect of Presentation Format on Judgment of Long-Range Time Intervals

**DOI:** 10.3389/fpsyg.2019.01479

**Published:** 2019-06-28

**Authors:** Camila Silveira Agostino, Yossi Zana, Fuat Balci, Peter M. E. Claessens

**Affiliations:** ^1^Department of Biological Psychology, Faculty of Natural Science, Otto von Guericke Universität Magdeburg, Magdeburg, Germany; ^2^Center for Mathematics, Computing and Cognition, Federal University of ABC, Santo André, Brazil; ^3^Department of Psychology, Koç University, Istanbul, Turkey

**Keywords:** temporal estimation, numerical estimation, personal events, power functions, model comparison

## Abstract

Investigations in the temporal estimation domain are quite vast in the range of milliseconds, seconds, and minutes. This study aimed to determine the psychophysical function that best describes long-range time interval estimation and evaluate the effect of numerals in duration presentation on the form of this function. Participants indicated on a line the magnitude of time intervals presented either as a number + time-unit (e.g., “9 months”; Group I), unitless numerals (e.g., “9”; Group II), or tagged future personal events (e.g., “Wedding”; Group III). The horizontal line was labeled rightward (“Very short” = >“Very long”) or leftward (“Very long” = >“Very short”) for Group I and II, but only rightward for Group III. None of the linear, power, logistic or logarithmic functions provided the best fit to the individual participant data in more than 50% of participants for any group. Individual power exponents were different only between the tagged personal events (Group III) and the other two groups. When the same analysis was repeated for the aggregated data, power functions provided a better fit than other tested functions in all groups with a difference in the power function parameters again between the tagged personal events and the other groups. A non-linear mixed effects analysis indicated a difference in the power function exponent between Group III and the other groups, but not between Group I and II. No effect of scale directionality was found in neither of the experiments in which scale direction was included as independent variable. These results suggest that the judgment of intervals in a number + time-unit presentation invoke, at least in part, processing mechanisms other than those used for time-domain. Consequently, we propose the use of event-tagged assessment for characterizing long-range interval representation. We also recommend that analyses in this field should not be restricted to aggregated data given the qualitative variation between participants.

## Introduction

Most studies of time perception have focused on the short time range (i.e., milliseconds to minutes – Grondin, 2010). Despite the importance of processing longer-range intervals for humans, investigations of time perception in the days-to-years range are less common. Virtually all inter-temporal decision-making tasks require the estimation of the magnitude of at least one long time interval at which the consequences of a choice would occur, and thus the psychophysics of time (i.e., the nature of mapping between objective [calendar] and subjective [perceived] time) has significant effects on the choice behavior in such scenarios. For instance, assume that intervals of 4 and 6 years are perceived as almost equally distant from now. A person has to choose between investing his/her money in mutual fund A or B. While fund A guarantees 40% nominal interest yield after 4 years, fund B guarantees 48% after 6 years. Equidistance perception of 4 and 6 year-long intervals might result in preference for investing in fund B, although a normative decision might suggest otherwise as the gain in the last 2 years is relatively low.

This paper concerns the estimation of subjective magnitude of long-range time intervals. We will refer to the relation between physical intervals and their internal representation as the psychophysical mapping function, and the psychometric function when we discuss the experimental measurement of this relationship. For obvious reasons, the direct measurement of the psychometric function of long-range time intervals is impractical. Thus, early studies of long time interval processing were based on the finding that the reaction time of the left-hand to small numbers is faster than to large numbers (Dehaene et al., 1993). Gevers et al. (2003, 2004) measured the Spatial Numerical Association of Response Codes (SNARC) effect, or the reaction time differences between right-handed minus left-handed responses, as a function of the day of the week or month of the year. They found a linear relation between the magnitude of the SNARC effect and the distance to the reference stimuli.

Arzy et al. (2008, 2009) formulated a different approach. They theorized that, when people consider points in time, they use a mental time line on which they can project personal life events. In order to test this view, they asked participants to indicate if an event occurred before or after a reference point in time. They found that reaction times were slower and responses less accurate for distant events in the past and future in comparison to the reference events. Reaction time decreased logarithmically with distance from the reference point. Thus, differently from previous SNARC effect studies, time perception follows a logarithmic function when measured based on mental time “self-projection.” It is noteworthy that both techniques are indirect in the sense that participants are required to discriminate between stimuli of different magnitudes rather than asked to estimate the magnitude of a specific time interval stimulus, and they rely on reaction times.

Instead, Faro et al. (2005, 2010) measured perception of long time intervals directly. In one study (Faro et al., 2005), participants were presented with pairs of historical events and asked to estimate the time between the two events in years. They did not, however, characterize the mapping functions. In another study, Faro et al. (2010) used a variant of the same procedure and reported a weak linear correlation between calendar and subjective time estimation without specifying the exact values. Kim and Zauberman (2009) and Zauberman et al. (2009) used a similar procedure, but presented time intervals textually in the form of a number and a time unit (e.g., “18 months”) and used a horizontal line as a scale for responding, i.e., the response was measured by cross-modality matching usually termed visual-analogue scale (VAS). In their study, participants indicated the line-length that corresponded to the subjective magnitude of the presented time interval. Their results suggest that subjective time follows a decelerating power function (see also Han and Takahashi, 2012; Bradford et al., 2014). This cross-modal matching method coupled with textual time-interval as stimulus allows for the estimation of the psychometric function for long-range time intervals. In order for a time interval presented in such a format to be understood accurately, one needs the premise that there is a linear relation between the textual-numerical presentation and its subjective magnitude. Thus, the influence of the perception of symbolic numerals (e.g., conventional Arabic numerals as a decimal numeral system) should be considered. The capacity to represent counts as analog quantities is usually referred to as the Approximate Number System (ANS). There is ample evidence that this representation follows a compressed form. For example, Dehaene et al. (2008), using a horizontal line for subjective estimation, found that adults perceived the quantity of dots in the range of 1–100 in a logarithmically decelerating manner.

Arabic numbers, on the other hand, are symbolic representations of quantities, i.e., they only acquire quantity information (its semantics) indirectly through learned association with analog non-symbolic quantities (Gallistel and Gelman, 1992). Siegler and Booth (2004) asked participants to estimate the magnitude of Arabic numerals. They used numbers in the 0–100 range and a line-length for responding. In the characterization of psychometric functions, they found a progressive shift from a highly decelerating logarithmic function for kindergarteners to a linear function for undergraduate students (see also Siegler and Opfer, 2003).

In a tentative account of the linear mapping of Arabic numerals and logarithmic mapping of analog quantities, Dehaene (1992) proposed the *triple-code model* in which symbolic representations of quantity are transmitted linearly but processed by the same system used to process analog quantities. However, there is also evidence that suggests a more complex mapping of symbolic numerals. For instance, Dehaene et al. (1990) asked participants to judge if two-digit numbers are larger than 65 and found that reaction times were logarithmically related to the numerical distance from the target. In another study, Schley and Peters (2014) used the line-length technique to estimate numerals in the 1–1000 range and found that participants could be classified as those presenting a linear or those presenting a nonlinear psychometric function. Hybrid models propose that estimation of the magnitude represented by symbolic numbers relies on a learned symbolic-number linear mapping and an innate concave ANS mapping (Schley and Peters, 2014; Peters and Bjalkebring, 2015).

A more general theory of cognitive processing of magnitudes in different domains, called A Theory of Magnitude (ATOM), was proposed in order to understand the relation between time, space, and quantities (Walsh, 2003; Bueti and Walsh, 2009). ATOM argues that temporal and non-temporal dimensions (e.g., space, numerosity, size, speed) share a common representational metric and that they are represented by an innate generalized magnitude system localized in the parietal cortex. Among ATOM’s direct predictions are the existence of a shared brain area for processing magnitudes of different domains and interference of magnitude estimation in one domain with the processing in other domains (e.g., Frassinetti et al., 2009). Moreover, ATOM predicts that processing in one domain influences processing in the other domains symmetrically, as all dimensions are processed by a single general-purpose analog magnitude system and no directionality or hierarchy between dimensions is specified (Martin et al., 2017). Supporting ATOM’s predictions, there is evidence of shared brain areas for processing space, time, and quantity (e.g., neural activity correlation in the inferior parietal cortex, especially the right intraparietal sulcus; for a review see Bueti and Walsh, 2009), as well as behavioral results in animals (e.g., rats could transfer their judgments across the numerical and temporal domain, Church and Meck, 1984) and humans (e.g., the above-mentioned SNARC-effect). However, evidence of linear mapping of numbers in adults, as opposed to logarithmic in children, and significant individual differences cannot be readily accounted for by the ATOM’s predictions. Furthermore, changes in one domain do not always affect the processing of another dimension, or there are asymmetries (Dormal et al., 2006; Karȿılar and Balcı, 2016). For instance, duration judgments were found not to be affected by surface or numerosity (Martin et al., 2017).

Developmental changes in the mapping functions of abstract numerals and asymmetry in the interference between different dimensions of stimuli are predicted by an alternative theory, the metaphor theory (Lakoff and Johnson, 1980, 1999). The metaphor theory proposes that people mentally represent abstract and complex knowledge as conceptual metaphors. A typical linguistic example would be “They moved the meeting forward 2 hours,” although it is not possible to perceive by our senses the “motion” of a meeting (Casasanto and Boroditsky, 2008). In western cultures, it is conventional to represent, in printed material such as graphs and comic strips, numbers and time as increasing horizontally from left to right. In view of the metaphor theory, time and space domains are independent at the beginning of development and become linked, asymmetrically, depending on individual experience, language, and culture (for a critical comparison of the two theories, see Winter et al., 2015). In support of the metaphor theory, there is evidence that people from different cultures and writing languages lay out time in space differently (Tversky et al., 1991; Fuhrman and Boroditsky, 2010; Anelli et al., 2018). For example, Tversky et al. (1991) asked participants to map events on a ruled-off square sheets of paper. They found that left-to-right organization was dominant for English speakers as compared to right-to-left for Arabic speakers (Hebrew speakers had no dominant preference), in correspondence with the respective direction of the language writing system. In an analogous task, the representation quantity was evaluated (ex. amount of sand). In contrast to the horizontal directionality of the representation of temporal properties, quantity properties showed no horizontal directionality: both right-to-left and left-to-right were equally used by participant of different speaking languages. However, other found directionality effect for numerical reasoning, which was modulated by culture and language. Directionality of numerical reasoning, just like temporal perception of events, was found to be modulated by culture and language (e.g., Dehaene et al., 1993; Bachtold et al., 1998; Zebian, 2005). French speaking participants showed left-to-right SNARC effect for large numbers and the inverse for small numbers (Dehaene et al., 1993; see also Bachtold et al., 1998 for similar results; for a review, see Toomarian and Hubbard, 2018).

Taken together, the empirical evidence and models suggest that symbolic numerals are perceived in a linear or concave shape in a way that varies between individuals. This leads to the question as to whether the Arabic numerals in the time interval stimulus type influence the resulting mapping function. This study aimed to bridge the empirical but theoretically critical gap in the literature by characterizing the psychophysical function that best mapped the subjective magnitudes onto objective long-range time intervals using different formats through which time intervals were presented. To this end, we tested participants in three different conditions; presentation of intervals as a number + time-unit (e.g., “9 months,” Group I), as unitless numerals (e.g., “9,” Group II), or as tagged future personal events (e.g., “graduation,” Group III). The effect of directionality of the time scale was also evaluated in the first two conditions under the assumption that different effects of scale direction indicate different cognitive processing.

Our results suggest that, when presented with time-intervals as numerals, people use, at least in part, knowledge from domains other than time to estimate magnitudes in this context. In addition, our results emphasize the importance of considering individual differences. Finally, we discuss the procedural implications of our findings.

## Experiment I: Numbers With Time Unit

### Materials and Methods

All participants were healthy undergraduate students that volunteered to take part of the experiment. Experimental protocols followed the conventional cross-modality line-length matching paradigm (Zauberman et al., 2009). In all three experiments, prior to the beginning of the first task, participants provided written and informed consent for their participation. The Research Ethics Committee at the Federal University of ABC approved all experimental protocols.

**FIGURE 1:**
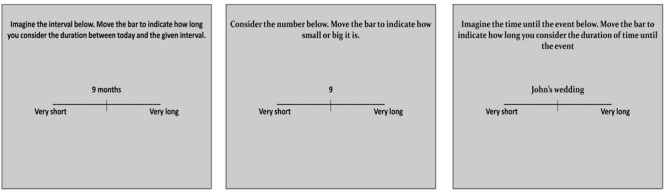
Experimental procedures. Instructions given to the participants in Experiment I (number and time-unit), II (number only), and III (personal event), respectively, from left to right panel. Experiments I and II were also performed with the labels at the extreme ends of the line presented in inverted position. Actual text was in Portuguese language.

In *Experiment I*, 18 participants (mean age 22.2 years, 10 women) were seated in an isolated laboratory room, at 70 cm from a computer monitor. The following instructions were presented on the screen, in Portuguese: “In this study you will be asked to indicate your subjective feeling of duration between today and many days in the future. Time intervals vary between 3 and 36 months. Please, read the instructions carefully and indicate your response.”^1^ After confirming that the participant had understood these instructions, the following text was displayed on the upper part of the screen (Figure 1, left panel): “Imagine the interval below. Move the bar to indicate how long you consider the duration between today and the given interval.”^2^ In each of two direction conditions, the time interval in months was presented below these instructions in the format, e.g., “15 months,” according to a random permutation of five repetitions from the set {3, 6, 9, 12, 15, 18, 21, 24, 27, 30, 33, 36}. Below the numeric time interval, a 180 mm line was presented with labels “very short” and “very long” at the extremes. In one configuration of the task, the labels “very short” and “very long” were placed at the left or right side, respectively (“forward” condition). In the other configuration, the order was reversed (“backward” condition). The presentation order of direction conditions was counterbalanced between participants. The initial position of the mouse cursor was always at the center of the line. Participants had to move the cursor to the right or left to arrive at the desired segment length and click the left mouse button to confirm their choice. The maximum response window was 10 s, after which a new trial was initiated. No-response trials were treated as missing values during statistical analysis. Each of the 12 time intervals was presented five times, totaling 60 trials per session. Four training trials with a random selection of intervals were presented at the beginning of the task to familiarize participants with the procedure; these data were not included in the analyses. The time intervals below 12 months were presented in a single digit format, e.g., “9,” rather than as double digits (“09”). The format used in the current study was chosen to be consistent with previous studies and thus allow for better comparison with their results (e.g., Zauberman et al., 2009).

#### Analysis

The evaluation of the explanatory power of different quantitative models and impact of experimental conditions was performed at three different levels of analysis. In all cases, linear, power, logarithmic, and logistic functions were fit to the data, in which each data point represented the average of five repeated measurements per participant, per condition. At a global analysis level, functions were fit to the data points (individual responses) from all the participants in a specific group using linear or nonlinear regression. At an individual level, the same analysis method was repeated, but on the data from each participant. Nonlinear mixed-effects modeling was used as an intermediate-level analysis, as it explicitly represents between-participant variation, while simultaneously estimating group means per condition. Model selection was conducted based on a Bayesian model selection approach. Amongst other advantages, Bayesian approach allows to gather probabilistic quantification of relative evidence in favor of all hypotheses, including the “null” hypothesis (Gallistel, 2009). It also includes, with a sound theoretical basis (Raftery, 1995; Wagenmakers, 2007), direct comparison of non-nested models, which is the case of the nonlinear models tested in this paper. As a general procedure, all four functions were fit with no restriction on their parameters. The preferred function was chosen according to the Schwarz weights ω*_i_* (Schwarz, 1978; Wagenmakers and Farrell, 2004):

$$\begin{array}{c} \Delta_{i}={\mathrm{BIC}}_{i}-minB\mathrm{IC}\\ \omega_{i}=\frac{\exp\left(-\Delta_{i}/2\right)}{\sum_{m=1}^{M}\exp\left(-\Delta_{m}/2\right)} \end{array}$$

where BIC is the Bayesian Information Criterion (Schwarz, 1978) and *M* is the number of models. The Schwarz weight for a model can be understood as its relative advantage in comparison with other models of a set, based on the posterior probabilities of all models involved. ω*_i_* assumes values from 0 to 1 and the sum of the weights of all functions is 1. The higher the weight of a specific function, the stronger the relative evidence in its favor. Since Schwarz weights are calculated using BIC, choosing according to highest Schwarz weight is identical to choosing according to lowest BIC, thus the selection criterion is actually the BIC and the weight a translation of relative evidence for a model. The Bayes Factor (*BF*) is another BIC-based measurement of relative evidence of models (Vandekerckhove et al., 2015). It is a useful measure to tell how much more probable one model is than another. However, in comparison with the ω*_i_*, it is restricted to pairwise comparisons. The value of the Bayes factor expresses how much one function is more likely than the other and is presented in this paper when a specific nonlinear function is compared to the linear alternative. The computation of a Bayes factor, in principle, takes into account a specific choice of a prior distribution over the model parameters; the exact value obtained depends on the choice of the prior. As a general approach, we calculated Bayes factors based on the Bayes information criterion. In practice, this approach can be considered one specific approximation to other possible Bayes factors, namely one that assumes that parameter priors are flat, and has the advantage of easy of computation. The Bayes Factor was therefore computed based on the BIC, as follows:

$$\begin{array}{c} \Delta BIC={\mathrm{BIC}}_{\mathrm{linear}}-{\mathrm{BIC}}_{\mathrm{nonlinear}}\\ BF=\exp\left(\Delta \mathrm{BIC}/2\right) \end{array}$$

A *BF* higher than 1 represents evidence in favor of the nonlinear function. As a reference, Bayesian factors differences between 3 and 20 constitute “positive” evidence while values between 20 and 150 constitute strong evidence in favor of the function with the lower factor. Any value above 150 can be taken as very strong evidence (Raftery, 1995).

All analyses were conducted in Matlab and The R Project for Statistical Computing, the latter with nonlinear mixed-effects estimation (*nlme)* package to obtain maximum-likelihood fits of non-linear models (Pinheiro et al., 2016).

### Results

**FIGURE 2:**
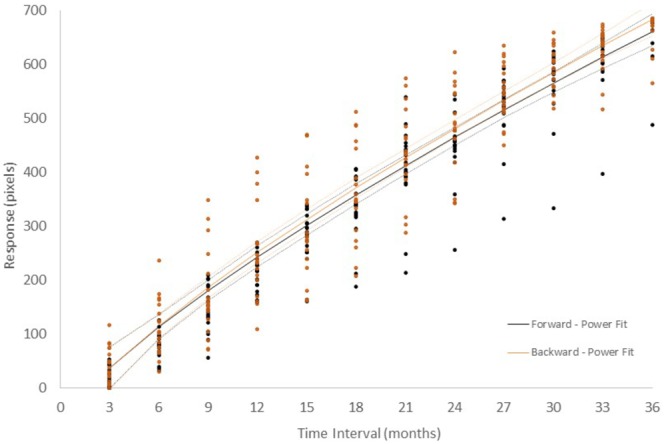
Number + time-unit interval magnitude estimation of the 18 participants in *Experiment I.* In the Forward configuration (black color), line-length was measured from left to right. In the Backward configuration (orange color), line-length was measured from right to left. Each dot represents the average of five measurements from an individual participant. Continuous lines represent the best fitting power functions, while dotted lines represent the upper and lower prediction bounds for the fitted functions with a confidence level of 95%. It should be noted that, while confidence intervals are usually associated with the distribution of data around a certain value, we show the prediction interval for a given confidence level, which is associated with the probability of the fitted function – without the random component representing inter-individual variation – being within an interval.

**FIGURE 3:**
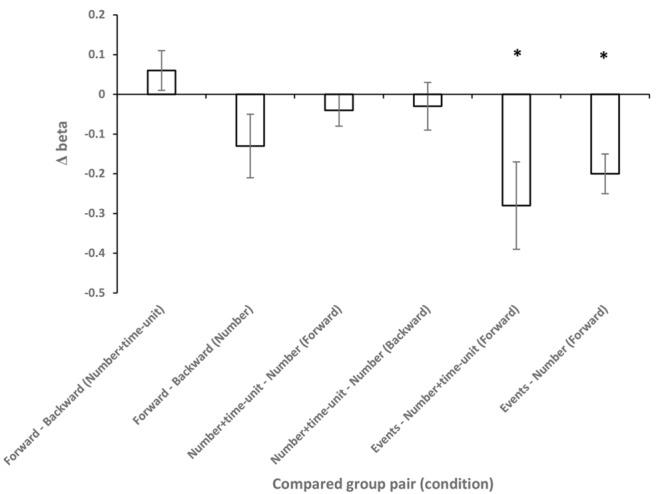
Results of the nonlinear mixed-effects power models for subsets of the data. The model was of the form c + αx^β^. The ordinate represents the difference between the β parameter of the fitted model for the tested pairs of conditions. The abscissa represents the tested group pairs: Direction (Forward-Backward) was tested in the Number + time-unit and Number conditions; stimulus type (Number + time-unit, Number, and Event) was tested in the Forward condition. Error bars represent standard error. Asterisks represent significant difference between groups (*p* < 0.05).

Figure 2 shows the responses in each configuration from *Group I – Number* + *time-unit* which performed *Experiment I* and in which participants were asked to indicate their estimate of the duration of the time interval. In the Forward configuration, in which the left extreme of the horizontal line was labeled “very short” and the right “very long,” participants picked a longer line length as the indicated time interval was increased. Similar results were found when the scale was inverted (Backward configuration). A linear, power, logarithmic and logistic regression analysis on the global data showed that, in all experimental conditions, over 88% of the variance was explained, with the exception of the logarithmic function that had lower values for all conditions (Supplementary Table 1). The power function had the highest posterior probability, as compared to the linear, logarithmic, and logistic functions, with ω*_i_* = 0.81, *BF* = 4.18 (positive evidence) and ω*_i_* = 0.88, *BF* = 8.24 (positive evidence), in the Forward and Backward conditions, respectively. One can say that, in the Forward condition, the data are 4.18 times as likely under the power function than they are under the linear function. Similar results were obtained when AIC (Akaike, 1974) was used instead of BIC. AIC is another popular information criterion that penalizes models less for complexity (number of free parameters). The estimates for the exponent of the power model were 0.77 and 0.75 in the Forward and Backward conditions, respectively. The effect of scale direction was evaluated using a nonlinear mixed-effects model (Figure 3 and Supplementary Table 2). The model was a power function of the form y = c + αx^β^, with the three parameters, α, β, and *c*, as fixed-effects dependent on the Direction condition, with additional Gaussian random-effect components to allow for individual effects, besides the error term. The predicted variable Y was the estimated line-length response on the original scale, i.e., in pixels. The result of this model fit showed no significant role for Direction (Figure 3, *t* = 1.17, *df* = 409, *p* = 0.24), thus the scale direction did not substantially influence the responses.^3^

## Experiment II: Numbers Only

Having tested participants and characterized their psychometric functions for long-range intervals with numeral-unit pairs in Experiment 1, we performed the same investigation in Experiment 2 with numbers only. The intervention aimed to test if the omission of time units has affected the nature of the psychometric function.

### Materials and Methods

Twenty volunteers (mean age 20.7 years, 7 women) participated in this experiment. Procedures were identical to those of *Experiment I*, with the exception of the references to time units. The following instructions were presented on the screen:^4^ “In this study you will be asked to indicate the MAGNITUDE (small or big) of numbers of values between 3 and 36. Read the instructions carefully and indicate your responses.” After the confirmation that the participant had understood these instructions, the following instructions were displayed on the upper part of the screen:^5^ “Consider the number below. Move the bar to indicate how small or big it is.” The number was presented below these instructions (Figure 1, middle panel). All analysis procedures were identical to those performed in *Experiment I*.

### Results

**FIGURE 4:**
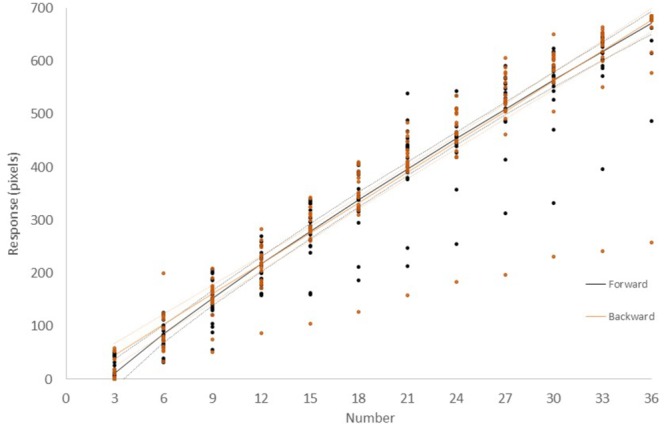
Number magnitude estimation of the 20 participants in *Experiment II.* In the Forward configuration (black color), line-length was measured from left to right. In the Backward configuration (orange color), line-length was measured from right to left. Each dot represents the average of five measurements from a single participant. Continuous lines represent the best fit power functions, while dotted lines represent the upper and lower prediction bounds for the fitted function with a confidence level of 95%.

Figure 4 shows the results for *Group II – Number* which performed *Experiment II* with numerical stimuli only and in which participants were asked to estimate the magnitude of numbers. Results were similar to those of *Experiment I*. A linear, power, logarithmic and logistic regression analysis on data aggregated across volunteers showed that in all cases, over 79% of the variance was explained, with the logarithmic function having the lowest values (Supplementary Table 1). The power function had the highest posterior probability, as compared to the linear, logarithmic and logistic functions, with ω_i_ = 0.81 in the Forward condition. However, in the Backward condition, the linear function was preferred with ω _i_ = 0.80. Bayes Factors were 4.35 and 4.40 (“positive evidence”) in the Forward and Backward conditions, respectively. However, when AIC was used instead of BIC, the power model was preferred. This outcome shows that, in the Backward configuration, the power model is preferred to the linear model when function complexity is less penalized. The estimates for the exponent (β) of the power model were 0.84 and 0.87 in the Forward and Backward configuration, respectively.

The effect of the scale direction was evaluated using a mixed-effects power model in the same manner as in Experiment I (Supplementary Table 2). The result of this model fit showed no significant effect of Direction (Figure 3, *t* = -1.72, *df* = 455, *p* = 0.08). However, the close to significant effect is in agreement with the finding of different best model fitting in the Backward and Forward condition at the global analysis level.^6^

## Experiment III: Personal Events

There was a large overlap between the presentation format across Experiment 1 and Experiment 2 as both contained a precise magnitude information presented in the form of Arabic numerals. In Experiment 3, we followed a novel route by referring to time intervals via personal events and tested if such a change in the presentation format would affect the psychometric function that maps objective long-range time intervals onto subjective magnitudes.

### Materials and Methods

Twenty-one volunteers participated in this experiment. Procedures were similar to those of Experiment I, with variations due to the different stimulus types. Participants were asked to list 20 events that are expected to happen in his or her personal life in the next month and up to 3 years in the future. Alongside the name, participants also wrote down the expected month and year of the expected personal event. Twelve events were chosen such that the spread of the time range was maximized. The experimental session took place after an interval of 2–7 days. Participants received the following instructions:^7^ “In this study, you will be asked to indicate your subjective feeling of the time interval duration between today and a certain future event. The events will occur in up to 36 months. Carefully read the instructions and indicate your answers.” After the confirmation that the participant had understood these instructions, the following instructions were displayed on the upper part of the screen (Figure 1, right panel):^8^ “Imagine the time until the event below. Move the bar to indicate how long you consider the duration of time until the event.” The name of one of the events was presented as the stimulus. After completing the task, participants were asked to say aloud the expected month and year of the presented events.

Participants were also asked to estimate the valence of the events in the range of a 1–5 Likert-type scale, 5 representing positive valence. For approximate matching of the valence of the events, events that were rated as negative, i.e., scored 1 or 2, were excluded from the analysis (Peters and Buchel, 2010). This exclusion had minor influence on the results, as only 12 events, across eight participants, met this exclusion criterion. Judgment of the duration of projected slides was previously found to be affected in a crossover interaction manner by valence and arousal levels: duration of negative slides was judged as shorter than that of positive slides for low-arousal stimuli, while the inverse was found for high-arousal stimuli (Angrilli et al., 1997). This might raise the concern that the low rate of cited negative events within this group could lead to more positive mood than their counterparts in the other two groups, thus biasing the results. However, there is some evidence that, in the particular case of the retrospective timing of remembered past events, the emotional content of said events and the participants’ mood at the moment of recall do not distort perceived duration (Grondin et al., 2014). Furthermore, any possible mood-changing effect of the interview was avoided by the period of 2-to-7 days between the listing of the events and the actual time-interval estimation task.

At the end of all trials, participants were asked to describe what they had to do in the task. All participants answered correctly, with two exceptions. One participant used the line scale to indicate the valence of the events while the other participant indicated the level of importance. Both participants were excluded from further analysis. All analysis procedures were identical to those performed in *Experiment I* and *II*, after a conversion that will be detailed below.

### Results

**FIGURE 5:**
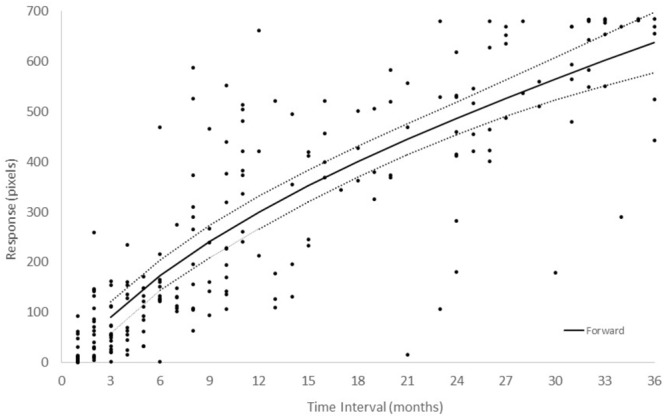
Event time interval estimations of the 19 participants in *Experiment III.* Only a Forward configuration was used with line-length measured from left to right. Each dot represents the average of five measurements from a single participant. The solid line represents the best fitted power function. The dotted lines represent the upper and lower prediction bounds for the fitted function with a confidence level of 95%.

**FIGURE 6:**
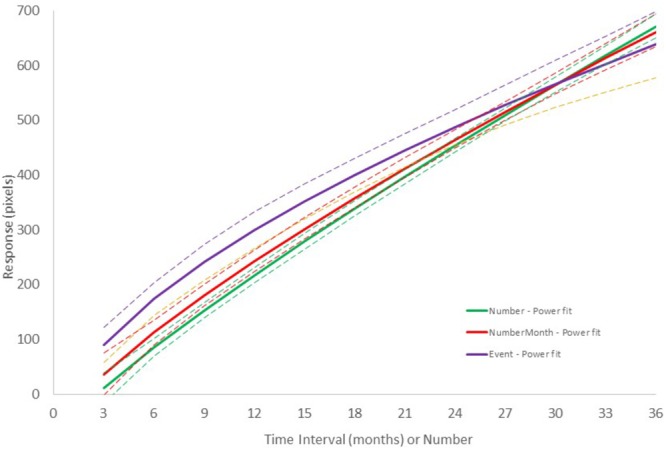
Comparison of the best fitting power functions for the Number + time-unit, Number, and Event experiments in the Forward configuration. Dashed lines represent the upper and lower prediction bounds for the fitted function of the corresponding color with a confidence level of 95%.

Figure 5 shows the results of *Group III – Events* for *Experiment III* with personal events as stimuli and in which participants were asked to indicate their feeling of the duration of time until the event in a Forward scale configuration. The conversion from event to time interval was made based on the month and year that the volunteers reported in the interview after the experiment (e.g., “Vacation to United States” – May, 2017). We calculated the number of months elapsed between the time of the task to the time of the event. Global level regression analysis using linear, power, logarithmic and logistic functions showed that, in all cases, over 68% of the variance was explained (Supplementary Table 1), the lowest found in this study. The power function had the highest posterior probability, with ω _i_ = 0.99. As expected from the ω _i_ value, the Bayes Factor was very high (*BF* = 140, “strong evidence”). Similar results were obtained when using AIC instead of BIC. The power model β parameter was as low as 0.58, indicating a more decelerated concave function. For comparison, Figure 6 presents the best-fit power models for the data in the three experiments in the Forward configuration.^9^

## Results Across Experiments I, II, and III

In all experimental conditions, global level analyses showed that power functions fit the data better, with the exception of the Number experiment in the Backward condition. Furthermore, it was observed that the best-fitting power function for the results of the Event experiment were more decelerated (β = 0.58). In this section we try to answer the following questions: (1) what is the degree of inter-individual variation? – and (2) did the manipulation of the stimulus type influence the responses, specifically the curvature of the psychometric function? In addition to the global level analysis, individual and mixed model analyses across experimental groups approach were adopted, where the mixed model refers to the use of methods that predict data at the group level as well as allowing for normal variation between individuals in parameter values.

### Individual Level Analyses

The following evaluation was designed to extend the testing of the working hypothesis that the curvature of the psychometric function depends on the use of numbers and time units taking an individual level approach. Using nonlinear regression analysis, power models were fit to the data of each participant (Table 1). Considering the β parameter as an estimator of the curvature of the psychometric function, we applied a linear mixed-effects model on the estimates of the corresponding parameter. Two fixed-effects variables were considered: Group to encode the experiment (Number + time-unit, Number) and Direction to encode the scale direction (Forward or Backward). The Event condition was not included, because the Direction variable has only one level (Forward) in this condition. Additional random-effect components were included to allow for inter-individual variation. The result of this model fit showed no statistically significant difference for Group (*t* = -1.36, *df* = 72, *p* = 0.17) or Direction (*t* = -0.045, *df* = 72, *p* = 0.96), thus the curvature of the psychometric function was not affected by including a time unit in the stimuli or by changing the scale direction.

**Table 1:** Power function (c + αx^β^), regressions of the individual data.

Experiment configuration	A	B	C	R^2^
Number + time-unit Forward	237.14 (928.76)	0.79 (0.38)	–318.27(705.57)	0.9845 (0.01)
Number + time-unit Backward	1409.85 (3747.45)	0.77 (0.44)	–1469.73(3832.98)	0.9876 (0.01)
Number Forward	51.93 (39.85)	0.88 (0.33)	–102.16(88.54)	0.9888 (0.01)
Number Backward	54.59 (48.50)	0.88 (0.39)	–89.02(117.30)	0.9782 (0.05)
Event Forward	5047.05 (15555)	0.61 (0.41)	–5348.41(15548)	0.7977 (0.17)

*Average values of the estimated model coefficients are given. R^*2*^ represents the average coefficient of determination. Values in parentheses represent standard deviation across participants.*

In order to compare the psychometric functions in the three experiments, including the Events, we defined Group as a factor with three experimental conditions: Number + time-unit, Number, and Events, all of them in the Forward configuration, and used linear mixed-effects model analysis to test the effect of Group on the β parameter. There was a significant difference (*t* = -2.79, *df* = 55, *p* = 0.007), indicating that the curvature of the psychometric function in the Event condition was higher than in the other conditions.

Finally, we extended the analysis to the individual subject level and evaluated the four alternative functions using regression analysis at an individual level. According to the model preference based on BIC or Schwarz weights, the power model did not account best for the data of more than 50% of the individuals in any experimental configuration (Table 2). In fact, considering all individuals in all experimental conditions, the linear function performed, similarly, to the power function. An extreme case is the Event configuration: Linear and logistic models explained better the data of 15 individuals as compared to only four by the logarithmic and power models.

**Table 2:** Number (and proportion in parentheses) of participants in each experimental condition whose data are better represented by a specific function based on BIC criteria.

Experiment configuration	Linear	Logarithmic	Logistic	Power^∗^	N
Number + time-unit Forward	6 (0.33)	1 (0.06)	5 (0.28)	6 (0.33)	18
Number + time-unit Backward	4 (0.22)	0 (0.00)	9 (0.50)	5 (0.28)	18
Number Forward	7 (0.35)	0 (0.00)	5 (0.25)	8 (0.40)	20
Number Backward	5 (0.25)	0 (0.00)	5 (0.25)	10 (0.50)	20
Event Forward	8 (0.42)	3 (0.16)	7 (0.26)	1 (0.05)	19
Average	(0.32)	(0.04)	(0.33)	(0.31)	

*^∗^In the case of two participants in the Number + time-unit Backward configuration and one participant in the Number Backward configuration, power function were initially selected based on the Schwarz weights criteria in which the four functions were compared. However, the Wald test performed afterward did not rejected the hypothesis that β = 1 and the linear function was chosen.*

### Mixed Model Analyses

We already evaluated the effect of the scale direction in Experiment I and II on the power model β parameter using a nonlinear mixed-effects model and reported that no significant effects of scale direction were found. The same analysis procedure was extended to verify the influence of stimulus type on the function parameters (Supplementary Table 2). There was no significant difference in the model parameters between the Number + time-unit and Number configuration group in the Forward (β: *t* = -1.09, *df* = 413, *p* = 0.27) and Backward (β: *t* = -0.61, *df* = 413, *p* = 0.53) condition. However, the estimates of α and β parameters of the model for the Event group were significantly different from those in the Number + time-unit (Forward; α: *t* = 1.99, *df* = 392, *p* = 0.04; β: *t* = -2.46, *df* = 392, *p* < 0.01) and Number (Forward; α: *t* = 2.57, *df* = 414, *p* < 0.01; β: *t* = -3.76, *df* = 414, *p* < 0.001) configuration. In summary, there was no evidence that the inclusion of the time unit in the stimulus label altered the parameters of the response model. However, the use of events, instead of Numbers or Numbers with a time unit, altered both the scaling (α) and curvature (β), with the function becoming more decelerated. Additionally, no evidence for the effect of the scale direction was found.

## General Discussion

We evaluated the effect of stimulus presentation and scale directionally on the psychophysical mapping function. In three experiments, participants were presented with either time-intervals in the format of “9 months”, numerical quantities in the format of “9” or time intervals indicated by the name of a personal future event such as “wedding.” In all experiments, participants used a horizontal line to indicate the estimated magnitude. The horizontal scale was labeled “Very short” and “Very long” from left to right, respectively; the first two experiments were also carried out with labels in inversed horizontal order. The common feature of the methodological approaches utilized in this paper is that they all measure how calendar times map onto the subjective long-scale timeline (internal metric representation) using a cross-domain transfer between subjective temporal distances and subjective spatial distances. The implied premises are that (a) the metric distances in the internal representation can be translated into metric distances in responding (akin to “amodal” magnitude estimation) and that (b) humans can transfer quantitative judgments across different magnitude domains (e.g., Balci and Gallistel, 2006). Another way to study the mappings between objective calendar times and the corresponding subjective temporal metrics would be to utilize a two alternative forced choice paradigm in which for instance participants are asked to make “too short” and “too long” judgments for different calendar times (e.g., Roach et al., 2011). In this case, information regarding certain biases in subjective metrics can be derived from the shape of psychometric functions (e.g., asymmetry) fit to these binary judgments.

When evaluated from a global perspective, considering only aggregated data, the responses to abstract numerals with and without a time unit were similar: data from both conditions were well explained by power functions and presented a similar deceleration rate, with the exception being the Number group in the Backward configuration which tended to favor the linear model instead. These results suggest that the psychometric function of abstract numerals follows a power model and that participants, when presented with time intervals that include numerals, are simply estimating the magnitude of the numerals. However, presenting time intervals with the indication of personal events, without the use of numerals, produced a different behavior. In this experimental condition, the power function clearly fit the data better in comparison with the other functions. Moreover, the power function fit in the Event condition was more decelerated (lower exponent) than the power function fit in the other conditions. Noteworthy is the lack of scale direction effect in both the Number and Number + time-unit conditions. A significant effect in one of the conditions, but not the other, would have indicated that different processing for numbers and numbers coupled with time units. Although the results do not reject such hypothesis, they do not support it.

These results partially corroborate previously published results. The psychometric functions of the number-only magnitude estimation were found to be linear or close to linear, a result that corroborates the psychophysical mapping of the magnitude of numerals in adults (Whalen et al., 1999; Dehaene et al., 2008). Power functions were also the best performing models when time intervals were presented with numerals, similar to those found in previous studies (e.g., Zauberman et al., 2009) in which an equivalent global level approach was used. However, it is noteworthy that, while a highly decelerated rate was registered previously with presentations of numerals coupled with a time unit (Kim and Zauberman, 2009), in the current study such rate was found only when stimuli were based on personal event tags, without the use of any abstract number or time unit. These results imply that people estimate time interval differently when presented with numbers and/or time units.

The effect of manipulating the stimulus type was more evident when individual differences were considered together with the general tendency of the participants in the experimental group. Nonlinear mixed-effects model analysis showed no difference in power function parameters between the experimental groups with number or number and time-unit, but also that both were different from the parameters of participants that were presented event-tagged time intervals. At a third, purely individual level analysis, the curvatures of the estimated psychometric power functions were compared and showed no difference between the experimental conditions with numbers and time-unit and numbers only, but a difference from the event-tagged condition. An evaluation of the best fitting model of each individual revealed that the power model is not the best overall representative of the data, being outperformed by other models in the Number+time-unit condition and particularly in the case of participants in the Event experiment. Results from the global and nonlinear mixed model analyses agree as to the power function better fitting in most experimental conditions, the minor influence of the time-unit and as to the influence of using event-tags in place of numbers and time units. These results are compatible with previous studies of estimation of number magnitude and time-intervals, although in the latter case the psychometric function was of higher deceleration rate. However, individual-level analysis revealed a different outcome. Power functions were outperformed by other models in four out of the five experimental conditions. Particularly in the event-tagged setup, the data of several participants were poorly fitted by power functions, and linear and logistic models were better suited. These results confirm the relevance of individual-level analysis in estimation of time-interval magnitude, although the comparison of global and individual level results should be always taken with caution given the differences in statistical power. In a recent study from our laboratory, using number and time-unit presentation, we showed that linear models fit 98% of the variance of the aggregated data as compared to approximately 90% in the current study (Agostino et al., 2017). However, the discrepancy can be explained by a normalization procedure in the former study that reduced substantially the variance. More importantly, findings from both studies emphasize the relevance of individual differences, with data from participants in the previous study split almost equally between linear and power models.

Time-interval presentation using references to events is not novel (Peters and Buchel, 2010), but to the best of our knowledge, it was not used previously for magnitude estimation and the results cannot be compared to those from previous studies. The responses in the Event condition were distinct from those in the other conditions, presenting a higher compression toward longer time intervals. This result, in addition to the earlier observed differences in best-fitted model distribution at the individual-level, suggests that, when no abstract numerals are presented, people apply different mechanisms or use a different strategy for time interval estimation.

One of the main findings of the current study is that people map the magnitude of stimuli presented as an abstract number and Number+time-unit in a similar way, while the magnitude of time intervals without the use of numerals are treated differently. This result is better explained by the metaphor theory, given that it posits a high weight on the influence of language and culture, and it can be argued that the relatively linear mapping of abstract numbers, a cultural concept, dominates cognitive processing. In their absence, as in the event-tagged time intervals, mapping could be free of such influences. The metaphor theory would also predict a scale directionally effect, given the assumed influence of spatial metaphors on representation of time and numbers. However, the lack of significant directionally effect in the current study does not support this view. The hypothesis that people rather focus on numbers than the accompanying units is corroborated by findings that cooperation level of participants in the prisoner’s dilemma game increased when the reward was increased from 3ȼ to 300ȼ, but not when it was increased from 3ȼ to 3$ (Furlong and Opfer, 2009). However, presentation format does not always alter time-preferences (Lukinova et al., 2019). In an intertemporal decision making study, time-intervals were indicated textually (ex. “64 days”) or by the amplitude of a pure tone. Discounted factors in the verbal and non-verbal conditions were positively correlated (*r* = 0.79).

The ATOM theory does not derive predictions on the effect of scale direction inversion. It would predict similar mapping functions for all three presentation conditions. Under this theory, the representation and processing of time and numbers are assumed to rely on the same magnitude representations and thus would carry the same metric structure (Walsh, 2003). This prediction was not confirmed, considering the differences in representation between the tagged-event condition and the two other conditions. However, we did not control the stimulus presentation for saliency, thus it can be argued that saliency differences are responsible for the different effects. In defense of the ATOM theory, it can also be argued that the use of the cross-modality line-length matching paradigm confounded the effects of processing in the numerosity and time dimension with processing in the spatial dimension. This is possible, in principle, but is unlikely. The processing in any dimension is composed of at least two mapping processes, stimulus-to-representation and representation-to-action. We chose to manipulate the stimulus dimension and kept the type of action constant. Any significant result could relate to both two processes, without being able to discriminate the locus of the effect. A similar approach was used by Dehaene et al. (2008), where the line-length matching paradigm was used to compare the magnitude estimation of dots (quantity), tones (sound) and numerals (abstract). Corroborating the results in this study, they found that western adult participants mapped symbolic numerals linearly, but logarithmically when quantities were presented in a non-symbolic fashion.

It is possible that, different from the numerical conditions, the “personal event” condition engaged the prospective memory system, namely the memory representations formed for a future event. One type of prospective memory is the time-based prospective memory that involves remembering to realize a certain event after a certain time and thus involve time estimation (Graf and Grondin, 2006). To this end, Block and Zakay (2006) have adapted their attentional model of interval timing to accommodate the temporal components of the time-based prospective memory, but the application of an internal-clock mechanism to very long intervals is not realistic due to the cognitive architecture and attentional resources (for a review see Waldum and Sahakyan, 2012). Furthermore, episodic memory is known to be used in making time estimations regarding future events (Roy et al., 2005). Briefly, these possible complex interactions between time estimates and memory systems might have underlain the differential effects observed in the “personal event” condition.

In summary, several conclusions can be drawn from the results of the current study. Amodal magnitude estimation of time intervals presented in the form of Number + time-unit, e.g., “15 months,” follows a slightly decelerated rate, similar to the psychophysical function observed with estimation of abstract numerals. Additionally, distinct psychophysical functions were found when people estimate the magnitude of time intervals presented without the use of abstract numerals. These results suggest that people do not necessarily invoke temporal domain mechanisms when presented with time intervals in the form of Number + time-unit and can be considered as evidence in support of the metaphor theory. The event-tagged time interval procedure can be argued to be more appropriate for measuring perception of long time intervals, given that it makes it more difficult for people to use a domain different from time, and can lead to the suggestion that time perception has a relatively high deceleration rate. Moreover, we have demonstrated that, in all experimental setups, individual differences are of major importance and the event-tagged paradigm is no exception. The source of the individual differences is not clear, and could reside in factors such as previous experience, numerical capacity, and type of personal events, but is a focus of ongoing research. In any case, psychophysical functions of time perception derived from aggregated data analysis, for example in models of inter-temporal decision making, should not be assumed to represent the perception of individuals in a non-discriminate way. Thus, the implications of the current work are both scientific and methodological: (a) through manipulation of the presentation format of the long-range time intervals we have shown that personal event tagged intervals are processed differently from those presented via numerals and (b) we have shown that there is substantial degree of individual variation in the function through which subjective magnitudes are mapped onto objective long-range time intervals.

## Ethics Statement

The Research Ethics Committee at the Federal University of ABC approved all experimental protocols.

## Author Contributions

CA contributed with ideas, development of codes for the experiments, recruitment and measurement of the volunteers, and executed the experiments, carried out statistical analyses and contributed to the manuscript writing and revision. YZ contributed with ideas, development of codes for the experiments, carried out statistical analyses, and contributed to the manuscript writing and revision. FB contributed with ideas and to the manuscript writing and revision. PC contributed with ideas, conducted statistical analyses, and contributed to the manuscript writing and revision.

## Conflict of Interest Statement

The authors declare that the research was conducted in the absence of any commercial or financial relationships that could be construed as a potential conflict of interest.

## Footnotes

1 “Nesse estudo, você será solicitado a indicar seu sentimento subjetivo da duração entre hoje e vários dias no futuro. Os dias podem variar entre 3 e 36 meses. Por favor, leia as instruções cuidadosamente e indique suas respostas.”

2 “Imagine o intervalo abaixo. Mova a barra para indicar quão longa você considera a duração entre hoje e o intervalo dado.”

3 Group and Direction effects were tested under the assumption that the power function most appropriately represents the data, based on the results of the aggregated data analysis. However, one can argue that, if individual differences would be considered, one of the other functions might be chosen. We verified this possibility taking a hierarchical approach and using non-linear mixed effects analysis, treating model parameters as individual and randomly distributed in the population, i.e., as random effects, to compare the four functions considered in this paper. Since there is inter-individual difference not only in parameters but also in which of the functions best fit the data, the models did not always converge without interventions – we allowed excluding up to two subjects per Group if necessary for convergence of any candidate in the model set, and optimizing parameters on a log rather than a linear scale, in effect testing different population distributions for the parameters. The results are in agreement with the fits on the aggregated data, with the power model outperforming the others when using either BIC or AIC as criterion. In Experiment I, the log Bayes factors for the power model versus the linear model were 23.99 and 54.99 for Forward and Backward configurations, respectively.

4 “Nesse estudo, você será solicitado a indicar a MAGNITUDE (pequeno ou grande) de números de valores entre 3 e 36. Leia as instruções cuidadosamente e indique suas respostas.”

5 “Considere o número abaixo. Mova a barra para indicar quão pequeno ou grande ele é.”

6 In a similar manner to the description in footnote 3, we applied nlme analysis on the data from Experiment II. The results are in agreement with the fits on the aggregated data, with the power model outperforming the others when using both BIC and AIC as criteria. In Experiment II, the log Bayes factors for the power model versus the linear model were 24.39 and 13.06 for Forward and Backward configurations, respectively.

7 “Nesse estudo, você será solicitado a indicar seu sentimento subjetivo da duração do intervalo de tempo entre hoje e algum evento pessoal futuro. Os eventos vão ocorrer em até 36 meses. Leia as instruções cuidadosamente e indique suas respostas.”

8 “Imagine o tempo até o evento abaixo. Mova a barra para indicar quão longa você considera a duração do tempo até o evento.”

9 In a similar manner to the description in footnote 3, we applied nlme analysis also on the data from Experiment III. The results are partially in agreement with the fits on the aggregated data, with the power model outperforming the others when using the AIC criterion, but the linear when using the BIC criterion. In Experiment III, the Bayes factor for the power versus the linear model was 0.025, and therefore favored the linear model.
