# Supplementary material for: Effect of Presentation Format on Judgment of Long-Range Time Intervals
Source: Front Psychol. 2019 Jun 28;10:1479. doi: 10.3389/fpsyg.2019.01479 (PMC6611061; doi:10.3389/fpsyg.2019.01479)
Supplement: Supplementary file 1 [file Table_1.pdf]

Supplementary Material

Effect of presentation format on judgment of long-range time intervals

Camila S. Agostino, Yossi Zana*, Fuat Balci, Peter M. E. Claessens

*** Correspondence:** Yossi Zana: yossi.zana@ufabc.edu.br

Table 1. Results of the model fits performed on the global data for the subsets of the three experimental groups. The four models are linear ($c + \alpha x$), power ($c + \alpha x^{\beta}$), logarithmic ($c + \alpha \log(x)$) and logistic ($\alpha/(1 + \exp(-(x - c)/\beta))$). Parameters are estimated across participants. SE indicates standard error of the parameter estimates.

Group subset	Model	Alpha (SE)	Beta (SE)	c (SE)	R^2
**Exp I Number+time-unit Forward**	Linear	18.69 (0.45)	-	8.76 (10)	0.89
	Power	46.37 (36.78)	0.77 (0.07)	-71.60 (36.78)	0.89
	logarithmic	258.54 (8.01)	-	-341.36 (22.89)	0.83
	Logistic	689.14 (24.05)	7.40 (0.51)	17.61 (0.73)	0.89
**Exp I Number+time-unit Backward**	Linear	19.28 (0.45)	-	7.92 (10.11)	0.89
	Power	50.10 (15.54)	0.75 (0.07)	-80.39 (37.95)	0.90
	logarithmic	266.93 (8.11)	-	-353.95 (23.17)	0.83
	Logistic	703.63 (22.99)	7.23 (0.48)	17.46 (0.68)	0.89
**Exp II Number Forward**	Linear	19.81 (0.32)	-	-27.83 (7.19)	0.94
	Power	37.11 (8.07)	0.84 (0.05)	-82.17 (23.14)	0.94
	logarithmic	272.07 (6.97)	-	-393.05 (19.92)	0.86
	Logistic	695.83 (18.03)	6.98 (0.35)	18.71 (0.52)	0.93
**Exp II Number Backward**	Linear	19.12 (0.45)	-	-11.37 (10)	0.88
	Power	31.35 (9.98)	0.87 (0.08)	-51.22 (30.67)	0.88
	logarithmic	261.55 (8.32)	-	-361.45(23.79)	0.80
	Logistic	697.26 (25.5)	7.3 (0.5)	18.62 (0.75)	0.88
**Exp III Event Forward**	Linear	17.65 (0.74)	-	52.44 (12.56)	0.71
	Power	90.11 (36.32)	0.58 (0.09)	-79.64 (54.48)	0.74
	logarithmic	175.46 (8.11)	-	-88.54 (19.11)	0.68
	Logistic	582.15 (24.74)	5.67 (0.66)	11.9 (0.89)	0.72

Table 2. Results of the nonlinear mixed-effects power models for subsets of the data. The model was of the form $c + \alpha x^{\beta}$, where $\beta = 1 + \beta'$. The effects of stimulus type (Number+time-unit, Number and Event) and Direction (Forward, Backward) were evaluated. Std. Error, DF, tStat and p stand for standard error, degrees of freedom, t-test statistic and two-tailed p-value, respectively.

Group subset	Fixed effects	Value	Std. Error	DF	tStat	p
**Group: Number+time-unit. Fixed effect levels: Forward & Backward direction: Direction (Forward & Backward)**	**α (Intercept)**	65.14	19.23	409	3.38	.00
	**α . Direction**	-11.65	11.05	409	-1.05	.29
	**β (Intercept)**	.67	.09	409	7.40	.00
	**β . Direction**	.06	.05	409	1.17	.24
	***c* (Intercept)**	-119.40	48.11	409	-2.48	.01
	***c* . Direction**	29.92	27.05	409	1.10	.26
**Group: Number. Fixed effect levels : Forward & Backward directions**	**α (Intercept)**	10.07	16.96	455	.59	.55
	**α .Direction**	20.06	12.63	455	1.59	.11
	**β (Intercept)**	1.02	.12	455	8.32	.00
	**β . Direction**	-.13	.08	455	-1.72	.08
	***c* (Intercept)**	-15.63	49.18	455	-.32	.75
	***c* . Direction**	-44.11	33.40	455	-1.32	.19
**Direction: Forward direction. Fixed effect Levels: Number+time-unit & Number subsets**	**α (Intercept)**	22.32	7.36	413	3.03	.00
	**α .Group**	3.60	4.92	413	.73	.46
	**β (Intercept)**	.97	.06	413	14.45	.00
	**β .Group**	-.04	.04	413	-1.09	.27
	***c* (Intercept)**	-71.00	36.06	413	-1.96	.04
	***c* .Group**	21.95	23.30	413	.94	.34
**Direction: Backward Fixed effect Levels: Number+time-unit & Number subsets**	**α (Intercept)**	32.92	15.78	413	2.08	.03
	**α .Group**	7.15	10.74	413	.66	.50
	**β (Intercept)**	.84	.09	413	8.92	.00
	**β .Group**	-.03	0.06	413	-.61	.53
	***c* (Intercept)**	-79.08	50.12	413	-1.57	.11
	***c* .Group**	2.69	32.95	413	.08	.93
**Direction: Forward Fixed effect Levels: Events & Number+time-unit subsets**	**α (Intercept)**	-86.47	64.01	392	-1.35	.17
	**α . Group**	58.17	29.38	392	1.99	.04
	**β (Intercept)**	1.45	.31	392	4.64	.00
	**β . Group**	-.28	.11	392	-2.46	.01
	***c* (Intercept)**	66.61	136.76	392	.48	.62
	***c* . Group**	-48.90	55.17	392	-.88	.37
**Direction: Forward Fixed effect Levels: Events & Number subsets**	**α (Intercept)**	-12.30	15.44	414	-.79	.42
	**α . Group**	32.43	12.58	414	2.57	.01
	**β (Intercept)**	1.19	.12	414	9.47	.00
	**β . Group**	-.20	.05	414	-3.76	.00
	***c* (Intercept)**	-7.18	43.57	414	-.16	.89
	***c* . Group**	-21.95	22.91	414	-.95	.33
